# A Network Pharmacology Study on the Molecular Mechanisms of FDY003 for Breast Cancer Treatment

**DOI:** 10.1155/2021/3919143

**Published:** 2021-02-06

**Authors:** Ho-Sung Lee, In-Hee Lee, Kyungrae Kang, Sang-In Park, Seung-Joon Moon, Chol Hee Lee, Dae-Yeon Lee

**Affiliations:** ^1^The Fore, 87 Ogeum-ro, Songpa-gu, Seoul 05542, Republic of Korea; ^2^Forest Hospital, 129 Ogeum-ro, Songpa-gu, Seoul 05549, Republic of Korea; ^3^Forestheal Hospital, 173 Ogeum-ro, Songpa-gu, Seoul 05641, Republic of Korea

## Abstract

Herbal medicines have drawn considerable attention with regard to their potential applications in breast cancer (BC) treatment, a frequently diagnosed malignant disease, considering their anticancer efficacy with relatively less adverse effects. However, their mechanisms of systemic action have not been understood comprehensively. Based on network pharmacology approaches, we attempted to unveil the mechanisms of FDY003, an herbal drug comprised of *Lonicera japonica* Thunberg, *Artemisia capillaris* Thunberg, and *Cordyceps militaris*, against BC at a systemic level. We found that FDY003 exhibited pharmacological effects on human BC cells. Subsequently, detailed data regarding the biochemical components contained in FDY003 were obtained from comprehensive herbal medicine-related databases, including TCMSP and CancerHSP. By evaluating their pharmacokinetic properties, 18 chemical compounds in FDY003 were shown to be potentially active constituents interacting with 140 BC-associated therapeutic targets to produce the pharmacological activity. Gene ontology enrichment analysis using g:Profiler indicated that the FDY003 targets were involved in the modulation of cellular processes, involving the cell proliferation, cell cycle process, and cell apoptosis. Based on a KEGG pathway enrichment analysis, we further revealed that a variety of oncogenic pathways that play key roles in the pathology of BC were significantly enriched with the therapeutic targets of FDY003; these included PI3K-Akt, MAPK, focal adhesion, FoxO, TNF, and estrogen signaling pathways. Here, we present a network-perspective of the molecular mechanisms via which herbal drugs treat BC.

## 1. Introduction

Breast cancer (BC) is a common female malignancy and a cause of mortality globally [1]. The genetic and epigenetic dysregulations in multiple cancer-associated genes and their key oncogenic signalings are implicated in the pathology of BC; these include the phosphoinositide 3-kinase- (PI3K-) Akt, tumor necrosis factor (TNF), forkhead box O (FoxO), erythroblastic leukemia viral oncogene homolog (ErbB), vascular endothelial growth factor (VEGF), hypoxia-inducible factor- (HIF-) 1, estrogen, p53, focal adhesion, and mitogen-activated protein kinase (MAPK) pathways [2–4]. Currently, chemotherapy, molecular-targeted therapy, and endocrine therapy are the major pharmacological approaches for BC treatment [5–10]. However, the long-term and frequent use of the aforementioned therapeutic drugs may induce toxic events that deteriorate quality of life of cancer patients, including gastrointestinal dysfunction, fatigue, peripheral neuropathy, immunosuppression and myelosuppression, cardiotoxicity, and osteoporosis [11–18]. In addition, the pharmacological efficacy of most molecular-targeted agents often falls short of expectations because of their limited capacity to therapeutically modulate the cancerous activities of various oncogenic cellular components [19]. These issues emphasize the need for anticancer agents that can pharmacologically regulate multiple oncogenes and pathways with safety. Herbal drugs are multicomponent therapeutics that elicit their pharmacological effects via multiple chemical compounds that target diverse disease-related genes, proteins, and pathways [20, 21]. Herbal medicines have attracted much attention due to their promising anticancer effects, reduced toxicities, and lower side effects [20, 21]. Previous clinical research studies have further shown that the use of herbal drugs can improve the tumor response, survival, health status, and quality of life of patients undergoing cancer therapy [22, 23].

FDY003 is an herbal formula composed of three herbal medicines [24, 25], namely, *Lonicera japonica* Thunberg (LjT), *Artemisia capillaris* Thunberg (AcT), and *Cordyceps militaris* (Cm), that have been reported to exert prominent anticancer effects in various cancer types [26–35]. It has been shown that FDY003 is a potent inhibitor of proliferation while promoting the apoptotic death of cancer cells and tumors [24, 25]. These activities involve regulation of key modulators of cell cycle and apoptosis, such as p53, p21, caspase-3, and Bcl-2-associated X protein (Bax) [24]. However, the molecular mechanisms of FDY003 against BC at the systemic level remain unclear.

Network pharmacology is a multidisciplinary research approach that uncovers complex disease mechanisms and can be used to formulate promising treatment strategies based on a systems perspective [36–39]. The interdisciplinary methodology integrates diverse scientific fields, such as medicine, pharmacology, network biology, systems biology, and computer science [36–39]. Network pharmacology has been demonstrated to be an efficient tool for the acquisition of comprehensive and systematic insights into the “multicompound, multitarget, multipathway” polypharmacological properties of herbal medicines, and it is extensively used to explore the active chemical compounds of herbal drugs and their therapeutic targets responsible for their pharmacological activities [36–39]. Network pharmacology investigates how associated systematic mechanisms are regulated through interactions among various key components and targets [36–39]. Here, we attempted to unravel the molecular mechanisms of anti-BC effects of FDY003 based on network pharmacology approaches.

## 2. Materials and Methods

### 2.1. Cell Culture

The MCF-7, MDA-MB-453, and MDA-MB-231 human BC cell lines were purchased from the Korean Cell Line Bank (Seoul, Korea). The cells were cultured in Dulbecco's Modified Eagle's Medium (DMEM, WELGENE Inc., Daegu, Korea) supplemented with 10% fetal bovine serum, 100 U/mL penicillin, and 100 *μ*g/mL streptomycin (Thermo Fisher Scientific Inc., Waltham, MA, USA). The cultured cells were maintained in a humidified atmosphere with 5% CO_2_ at 37°C.

### 2.2. Preparation of FDY003 Herbal Formula

The preparation of FDY003 was conducted as previously described [25]. In brief, the raw herbal constituents of FDY003 were obtained from Green Myeong-Poom Pharm. Co., Ltd. (Namyangju, Korea). The dried plant materials of LjT (4.16 g), AcT (6.25 g), and Cm (6.25 g) were ground, added to 70% ethanol (500 mL), and subjected to reflux extraction at 80°C for 3 h. Then, the herbal extract was filtered through a 1 *μ*m pore filter (Hyundai Micro, Seoul, Korea) and successively purified with 80% and 90% ethanol. The resulting solution was lyophilized at −80°C. The samples were stored at −20°C and then dissolved in distilled water before the experiments.

### 2.3. Cell Viability Assay

The cell viability assay was performed following the previous procedures [25]. 3-(4,5-Dimethylthiazol-2-yl)-2,5-diphenyltetrazolium bromide (MTT) was obtained from Sigma-Aldrich Inc. (St. Louis, MO, USA). Cell viability was measured using the MTT assay. The cells were seeded in 96-well plates (5.0 × 10^4^ cells per well) and then treated with the indicated drugs for 72 h in a 5% CO_2_ incubator at 37°C. Subsequently, MTT solution (200 *μ*L) was added to each well, and the cells were further incubated for 2 h. Thereafter, the resulting formazan crystals were dissolved in dimethyl sulfoxide, and the absorbance was read with an Epoch 2 Microplate Spectrophotometer at 550 nm (BioTek, Winooski, VT, USA).

### 2.4. Exploration of Active Chemical Compounds

Comprehensive information on the phytochemical components of FDY003 was integrated from traditional Chinese medicine systems pharmacology (TCMSP) and anticancer herbs database of systems pharmacology (CancerHSP) databases [40, 41]. To determine the bioactive compounds of FDY003, we assessed the key absorption, distribution, metabolism, and excretion (ADME) pharmacokinetic parameters (i.e., oral bioavailability (OB), drug-likeness (DL), and Caco-2 permeability) of chemical constituents obtained from the TCMSP database [40]. OB, a pivotal consideration in drug development, is a measurement of the rate, fraction, and extent of an orally administered drug that reaches the expected site of drug action [40, 42]. Caco-2 permeability is a parameter widely used for the evaluation of the intestinal absorption rate and extent of a given substance using Caco-2 human intestinal epithelial cells [40, 43–45]. In general, drug molecules with a Caco-2 permeability less than −0.4 are considered not permeable across the epithelium of intestines [40, 46, 47]. DL is an indicator that is used to assess whether a compound has the potential to be developed into a drug with respect to its physical and chemical properties; it is calculated based on the Tanimoto coefficient and relevant molecular descriptors [40, 48]. A chemical compound is considered active if it meets the following criteria: OB ≥ 30%, Caco-2 permeability ≥ −0.4, and DL ≥ 0.18 [37, 40, 49, 50].

### 2.5. Target Identification for the Active Compounds

Molecular targets of the bioactive compounds of FDY003 were determined using comprehensive information regarding chemical-protein interactions obtained from various relevant databases, including Search Tool for Interactions of Chemicals (STITCH) 5 [51], SwissTargetPrediction [52, 53], PharmMapper [54], and Similarity Ensemble Approach (SEA) [55]. We also used in silico models, such as systematic drug targeting tool (SysDT) [56] and weighted ensemble similarity (WES) algorithm [57], for target identification according to previously described procedures [58–63]. Human genes/proteins related to the pathology of BC were obtained from the following databases: Therapeutic Target Database (TTD) [64], GeneCards [65], Comparative Toxicogenomics Database (CTD) [66], DisGeNET [67], Human Genome Epidemiology (HuGE) Navigator [68], Online Mendelian Inheritance in Man (OMIM) [69], Pharmacogenomics Knowledgebase (PharmGKB) [70], and DrugBank [71].

### 2.6. Network Construction

The herbal medicine-bioactive compound (H-C) and bioactive compound-target (C-T) networks were generated by connecting the three herbal constituents of FDY003 with the bioactive compounds and the bioactive compounds with the targets. The target-pathway (T-P) network was generated by connecting the targets with relevant biological pathways. The protein-protein interaction (PPI) network was generated based on the interactions between the targets (confidence score ≥ 0.9) using the STRING database [72]. Network visualization and analysis were performed with Cytoscape [73]. In the presented data, nodes indicate the herbal constituents, active chemical constituents, targets, or pathways, and edges (or links) indicate their interactions [74]. The degree indicates the number of edges of a node in a network [74].

### 2.7. Contribution Index Analysis

The contribution of chemical compounds to the pharmacological activity of FDY003 was analyzed using a contribution index (CI) [50] that can be calculated using the following formula:

(1) $$\begin{array}{c} \text{NE}\left(j\right)=\sum_{i=1}^{n}d_{i},\\ \text{CI}\left(j\right)=\frac{c_{j}\times \text{NE}\left(j\right)}{\sum_{i=1}^{m}c_{i}\times \text{NE}\left(i\right)}\times 100\%, \end{array}$$

twhere NE indicates the network-based efficacy, *n* indicates the number of targets of chemical component *j*, *d*_*i*_ indicates the number of links of target *i* of chemical component *j*, *m* indicates the number of chemical components, and *c*_*i*_ (or *c*_*j*_) indicates the number of previous literatures containing the terms “breast cancer” and the common name of chemical component *i* (or *j*) in their title or abstract that were retrieved from the PubMed (https://pubmed.ncbi.nlm.nih.gov/). If the sum of the highest CIs is greater than 85%, the compounds with those CIs are considered the major contributors, as previously suggested [50].

### 2.8. Functional Enrichment Analysis

Gene ontology (GO) enrichment analysis was performed using g:Profiler [75], and pathway enrichment analysis was carried out with Kyoto Encyclopedia of Genes and Genomes (KEGG) databases [76].

## 3. Results

### 3.1. Anticancer Properties of FDY003 against Breast Cancer

To investigate whether FDY003 exerts therapeutic effects on BC cells, we treated MCF-7 (an estrogen receptor-positive human BC cell line), MDA-MB-453 (a human epidermal growth factor receptor 2- (HER2-) positive human BC cell line), and MDA-MB-231 (a triple-negative human BC cell line) cells with FDY003 for 72 h and observed their responses. We found that FDY003 repressed the viability of MCF-7 (IC_50_ = 242.90 *μ*g/mL), MDA-MB-453 (IC_50_ = 156.01 *μ*g/mL), and MDA-MB-231 (IC_50_ = 197.56 *μ*g/mL) cells (Supplementary Figure S1), suggesting that the herbal medicine possesses anti-BC properties.

### 3.2. Chemical Components of FDY003

The chemical compounds that are present in FDY003 were obtained from the comprehensive databases associated with herbal medicine such as TCMSP and CancerHSP [40, 41]. Accordingly, 323 compounds were retrieved for FDY003 after removing duplicates (Supplementary Table S1).

### 3.3. Active Chemical Compounds in FDY003

Compounds whose pharmacokinetic parameters met the following criteria were considered active as described in Section 2.4: OB ≥ 30%, Caco-2 permeability ≥ −0.4, and DL ≥ 0.18 [49, 50]. A number of compounds not satisfying the aforementioned criteria were also considered bioactive because they were present in large amounts in herbal medicines and were known to have potent pharmacological efficacy. As a result, we obtained 20 active compounds for FDY003 (Supplementary Table S2).

### 3.4. Targets of Active Chemical Compounds in FDY003

**Figure 1:**
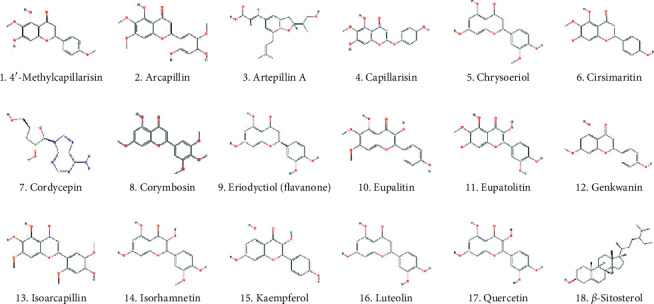
The active chemical compounds of FDY003.

We used comprehensive chemical-protein interaction data obtained from various relevant databases, including STITCH [51], SEA [55], SwissTargetPrediction [52, 53], and PharmMapper [54] to explore the molecular targets for the bioactive chemical components in FDY003. In addition, in silico models, such as SysDT [56] and WES algorithms [57], were used for the target exploration based on previously described procedures [58–63]. Consequently, we obtained 196 targets for the 18 active compounds (i.e., 4′-methylcapillarisin, arcapillin, artepillin A, capillarisin, chrysoeriol, cirsimaritin, cordycepin, corymbosin, eriodyctiol (flavanone), eupalitin, eupatolitin, genkwanin, isoarcapillin, isorhamnetin, kaempferol, luteolin, quercetin, and *β*-sitosterol) in FDY003 (Figure 1 and Supplementary Table S3). No interacting targets were retrieved for the compounds “loniceracetalides B_qt” and “demethoxycapillarisin.”

### 3.5. Network Pharmacology Study on the Molecular Mechanisms of FDY003

**Figure 2:**
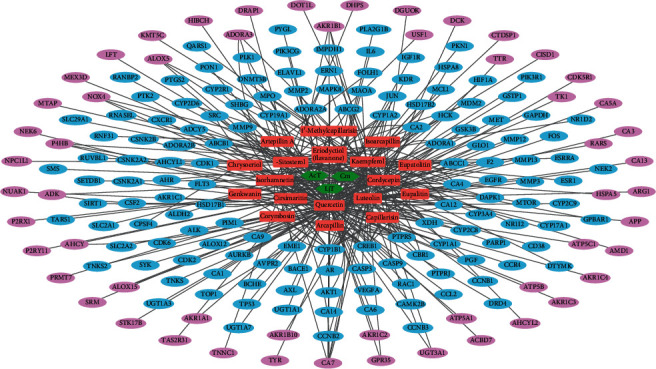
Herbal medicine-active chemical compound-target network of FDY003. Green hexagons, herbal medicines; red rectangles, active chemical compounds; blue ellipses, BC-associated targets; purple ellipses, non-BC-associated targets.

**Figure 3:**
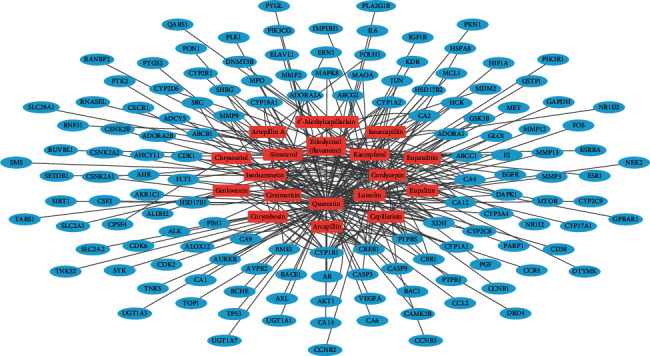
Active chemical component-target network of FDY003. Red rectangles, bioactive chemical components; blue ellipses, breast cancer-associated targets.

To conduct network pharmacology analysis of the molecular mechanisms of FDY003 against BC, we first generated an herbal medicine-bioactive compound-target (H-C-T) network of the herbal formula by linking the herbal medicines with their bioactive chemical components and the components with the targets (Figure 2). The resulting H-C-T network contained 217 nodes (3 herbal medicines, 18 active chemical components, and 196 targets) and 354 edges (Figure 2). In addition, to obtain insight into the BC-associated pharmacological features of FDY003, we constructed a C-T network (158 nodes with 254 edges) by connecting the bioactive chemical components with the BC-associated targets (Figure 3 and Supplementary Table S3). The quercetin, luteolin, kaempferol, cordycepin, eriodyctiol (flavanone), isorhamnetin, and *β*-sitosterol exhibited the highest degrees (Figure 3 and Supplementary Table S3), implying that they are essential for the mediation of the anticancer effects of FDY003 against BC. Furthermore, 42 BC-associated targets interacted with two or more compounds (Figure 3 and Supplementary Table S3), supporting the polypharmacological characteristics of FDY003.

**Figure 4:**
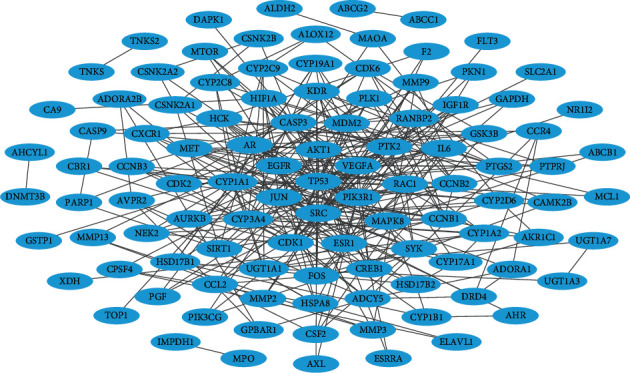
Protein-protein interaction network for breast cancer-associated targets of FDY003. Nodes refer to the breast cancer-related targets of FDY003.

To investigate the interactive associations among the targets, we built a PPI network (106 nodes and 315 edges) consisting of the BC-associated therapeutic targets of FDY003 (Figure 4). Subsequently, we explored the existence of hubs (i.e., nodes with relatively high degrees that tend to play prominent roles in the cellular processes in a network) [77, 78]. In the analysis, we defined hubs as nodes with degrees equal to or greater than twice the mean node degree [79, 80]. Among the BC-associated targets of FDY003, TP53, SRC, PIK3R1, VEGFA, AKT1, EGFR, CYP1A1, CYP3A4, JUN, CDK1, and ESR1 were hub nodes (Figure 4), suggesting that the nodes act as important targets mediating the therapeutic effects of FDY003 against BC cells. Loss of function of p53 (encoded by *TP53*) due to genetic alterations has been shown to drive the tumorigenesis, progression, and metastasis of BC; p53 expression has been reported to be a potential prognostic indicator for BC patients [81–89]. The dysregulation and elevated activity of the kinase Src (encoded by *SRC*) is frequently observed in multiple human malignancies, including BC, and it promotes the invasion, metastasis, migration, and proliferation of BC cells [90–94]. The expression and activity of *SRC* or *PIK3R1* are highly upregulated in malignant breast tumor tissues and have been correlated with decreased survival of BC patients [95–97]. VEGF-A (encoded by *VEGFA*) is a crucial regulator in the proliferation, angiogenesis, and metastatic behavior of BC cells, and it confers resistance against chemotherapy [98–101]. The overexpression or hyperactivation of AKT (encoded by *AKT1*), epidermal growth factor receptor (EGFR; encoded by *EGFR*), or c-Jun (encoded by *JUN*) promotes various cancerous processes, including proliferation, growth, survival, invasion, and migration of BC cells and is further related to the poorer clinical outcomes of BC patients [102–127]. Such targets have been implicated in reduced drug sensitivity of cancer cells to chemotherapeutics; therefore, targeting them could improve the therapeutic efficacy of chemotherapy and radiotherapy in BC [104–106, 109, 111–113, 117, 119, 123, 125, 126, 128–131]. Cytochrome P450 1A1 (encoded by *CYP1A1*) and cytochrome P450 3A4 (encoded by *CYP3A4*) are modulators of estrogen metabolism, and their activities are involved in the cancerous processes of BC cells [132–139]. Genetic polymorphism and expression of CYP3A1 or CYP3A4 in breast tumor tissues have been reported to be potentially useful factors for the prediction of treatment responses to chemotherapy [140, 141]. CDK1 (encoded by *CDK1*) functions as a crucial regulator in cell cycle progression, and its dysregulation leads to aberrant proliferation of BC cells [142]. Previous studies have indicated that CDK1 activity may act as a prognostic indicator in BC, and CDK1 targeting can increase chemotherapeutic efficacy [143–147]. Abnormal activity of estrogen receptor *α* (encoded by *ESR1*) is considered primarily responsible for tumorigenesis and progression of BC, and the receptor is the most promising therapeutic target [134–139].

To assess the contribution of the chemical components to the pharmacological effects of FDY003, we calculated CIs for the individual active compounds (Section 2.7) [50, 148]. As a result, quercetin and luteolin had the highest CIs with a sum of 91.83% (Supplementary Figure S2), which suggests that the two active components are key factors contributing to the FDY003 anticancer properties in BC treatment.

Overall, the results of the analyses above facilitate the identification of the polypharmacological mechanisms of FDY003 activity against BC.

### 3.6. Functional Enrichment Analysis for the FDY003 Network

To investigate the biological roles of the BC-related targets of FDY003, we carried out GO enrichment analysis for the targets. These targets were enriched in GO terms for the modulation of biological processes, involving cell proliferation, cell cycle progression, and cell apoptosis (Supplementary Figure S3), highlighting the molecular properties of FDY003 activity.

**Figure 5:**
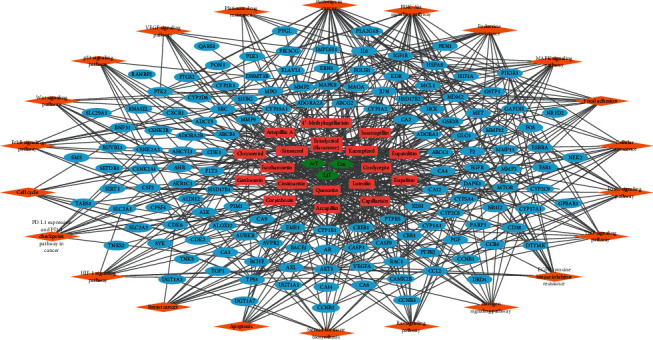
Herbal medicine-compound-target-pathway network of FDY003. Green hexagons, herbal medicines; red rectangles, bioactive chemical components; blue ellipses, breast cancer-related targets; orange diamonds, signaling pathways.

The aberrant activities of oncogenic cellular signalings are known to be responsible for cancer development and progression [149]. To this end, we next carried out pathway enrichment analysis for its BC-related targets (Figure 5 and Supplementary Figure S3). We found that the following diverse pathways, which importantly function in the tumorigenesis and progression of BC, were significantly enriched with the FDY003 targets: “Pathways in cancer,” “PI3K-Akt signaling pathway,” “Endocrine resistance,” “MAPK signaling pathway,” “Focal adhesion,” “Cellular senescence,” “FoxO signaling pathway,” “TNF signaling pathway,” “EGFR tyrosine kinase inhibitor resistance,” “Estrogen signaling pathway,” “Ras signaling pathway,” “Steroid hormone biosynthesis,” “Apoptosis,” “Breast cancer,” “HIF-1 signaling pathway,” “PD-L1 expression and PD-1 checkpoint pathway in cancer,” “Cell cycle,” “ErbB signaling pathway,” “Wnt signaling pathway,” “p53 signaling pathway,” “VEGF signaling pathway,” and “Platinum drug resistance” (Figure 5 and Supplementary Figure S3). The dysregulation of PI3K-Akt, MAPK, focal adhesion, and Ras signaling pathways promotes diverse cancerous cell processes, including the uncontrolled cell proliferation, invasion, migration, survival, metastasis, and angiogenesis of BC cells [3, 126, 150–154]. Abnormalities of crucial cellular function, such as senescence, apoptosis, and cell cycle, are the important pathological processes of BC [155–160]. The TNF signaling pathway is a mediator of the inflammatory process, and its activity is closely linked with the progression, metastasis, and poor prognosis of BC [161, 162]. The estrogen signaling pathway functions as the most critical regulator of tumor initiation and malignant progression in BC, and therapeutic modulation of its activity serves as a primary treatment strategy [163–167]. Previous studies have suggested that expression of programmed death-ligand 1 (PD-L1) serves as a prognostic factor for the survival of patients with BC and that inhibition of the programmed cell death protein 1 (PD-1)/PD-L1 pathway can enhance antitumor responses [168–172]. The HIF-1 and Wnt signaling regulate various cellular behaviors, involving cell proliferation, metastasis, and stem cell-like characteristics in BC cells [173–180]. The p53 signaling pathway exerts tumor-suppressive activity associated with cell cycle arrest, apoptosis, and cellular senescence, and loss of function of its key pathway components has been implicated in the carcinogenesis of BC and is a negative prognostic factor for patient survival [85, 181]. The VEGF signaling pathway plays a protumoral role by increasing angiogenesis, thus promoting the survival, migration, and invasion of BC cells [101, 182]. In addition, resistance to platinum-based drugs, endocrine therapy, and EGFR signaling inhibitors are major obstacles in BC treatment [183–189].

We further analyzed the functional associations among FDY003 targets using GeneMANIA [190], an algorithm useful for the analysis of biological functions of cellular components based on extensive network integration. Among the BC-associated targets of FDY003, 38.32% and 33.65% of them tended to be coexpressed and physically interacting, respectively (Supplementary Figure S4), suggesting that they have similar biological roles and functions.

Together, the results above suggest that FDY003 exerts the pharmacological activity by targeting diverse BC-associated oncogenic signaling pathways and the modulation of relevant biological functions.

## 4. Discussion

BC is a common cancer type and ranks as the leading cause of death among women globally [1]. Herbal medicines are attracting considerable attention for potential applications in cancer treatment owing to their high anticancer activities, reduced toxicity, and minimal adverse effects [21]. Based on a network pharmacology analysis, we explored the molecular mechanisms of the therapeutic effects of FDY003 for BC. (i) FDY003 exhibited anticancer effects on human BC cells (Supplementary Figure 1). (ii) Eighteen potentially active compounds (i.e., 4′-methylcapillarisin, arcapillin, artepillin A, capillarisin, chrysoeriol, cirsimaritin, cordycepin, corymbosin, eriodyctiol (flavanone), eupalitin, eupatolitin, genkwanin, isoarcapillin, isorhamnetin, kaempferol, luteolin, quercetin, and *β*-sitosterol) present in FDY003 may interact with 140 BC-associated therapeutic targets and induce the pharmacological activity of the herbal drug (Figures 1–4). (iii) GO terms for the modulation of cellular processes were significantly enriched for the FDY003 targets, including cell proliferation, cell cycle process, and cell apoptosis (Supplementary Figure 3). In addition, (iv) diverse pathways that play key roles in BC pathology were enriched for the targets that included PI3K-Akt, MAPK, focal adhesion, FoxO, TNF, and estrogen signaling pathways (Figure 5 and Supplementary Figure 3).

The FDY003 constituents have been reported to exert inhibitory effects against BC. AcT inhibited the proliferation but induced the death of BC cells [191]. Cm has been previously demonstrated to reduce the migratory and proliferative capacities of BC cells and to stimulate apoptosis by promoting caspase activation and Akt inactivation [29, 35, 192, 193]. Cm also has immunomodulatory properties that can inhibit the growth of breast tumors [194]. Capillarisin exhibits its anticancer effects by attenuating the invasive and proliferative properties of BC cells [195]. Chrysoeriol treatment has been reported to promote apoptosis and cell cycle arrest and further repress the invasion, proliferation, and migration of BC cells [196, 197]. Cirsimaritin inhibits proliferation and angiogenesis via the downregulation of VEGF, Akt, and extracellular signal-regulated kinase (ERK) [198]. Cordycepin is a potent inhibitor of the invasion and proliferation of BC cells while inducing their apoptosis through the regulation of MAPK and caspase-dependent pathways [199–203]. Cordycepin has also been shown to function as a radiosensitizer that can enhance the efficacy of radiotherapy toward BC cells [204]. Genkwanin modulates the activities of CYP1 enzymes and PI3K/Akt/mammalian target of rapamycin (mTOR) pathways, thereby suppressing proliferation and inducing apoptosis of BC cells [205–207]. Isorhamnetin exerts the anticancer activity against BC cells by inhibiting their proliferative and invasive abilities [208–210]. *β*-Sitosterol activates key apoptotic pathways, including Fas and caspase signaling pathways, and reduces the viability of BC cells [211–214]. Furthermore, *β*-sitosterol has been demonstrated to elevate the pharmacological effectiveness of tamoxifen, a selective estrogen receptor modulator that is extensively applied in clinical practice [215]. Kaempferol, luteolin, and quercetin stimulate apoptotic cell death but inhibit cell processes, including proliferation, cell cycle progression, angiogenesis, migration, invasion, metastasis, and cancer stemness; such effects occur via the regulation of important BC-associated pathways such as the Akt, caspase, EGFR, estrogen, HER2, MAPK, insulin-like growth factor (IGF)-1, Notch, and Wnt signaling pathways [216–266]. The three chemical compounds have also been shown to sensitize BC cells to various anticancer drugs, including cisplatin, docetaxel, doxorubicin, lapatinib, paclitaxel, rapamycin, sorafenib, tamoxifen, topotecan, and vincristine [267–286]. For instance, luteolin can synergistically enhance the growth-suppression and apoptosis-inducing activities of the anticancer agent celecoxib against BC cells by blocking the activation of oncogenic Akt and ERK signaling [271, 272]. The combined treatment of quercetin with kaempferol or luteolin has synergistic antiproliferative effects that are greater than those of either treatment exclusively [287, 288]. The risk of BC incidence showed a tendency to be lower in women with higher quercetin intakes [289].

Pharmacologic effects of FDY003 in cancer cells have been previously reported [24, 25]. FDY003 has been reported to exert its anticancer effects through the regulation of the activities of key mediators of apoptosis and cell cycle progression; these involved Bax, caspase-3, p21, and p53 that induce apoptosis while suppressing the proliferative and survival capacities of cancer cells [24, 25]. Treatment with the herbal formula further inhibited tumor growth in xenograft mice bearing human cancer cells [24], suggesting in vivo therapeutic effects against cancer. Contrary to the treatment with irinotecan, a clinically used cytotoxic chemotherapeutic agent [290], body weight loss (a parameter used to evaluate the potential toxicity of drug treatments in animal experiments) did not occur in FDY003-administered xenograft mice [24], suggesting tolerability of the herbal drug as well as its antitumor activity. Future experimental studies should (i) investigate the pharmacological effects of FDY003 in diverse types of cancer, (ii) explore the mechanisms underlying the anticancer activity of the herbal formula such as its immunomodulatory effects, and (iii) evaluate the anticancer effectiveness and safety of FDY003 combined with other widely used therapeutic approaches (i.e., chemotherapy, endocrine therapy, and targeted molecular therapy). Such studies would facilitate the development of safer and more effective herbal medicine-based strategies for BC treatment.

## 5. Conclusions

We explored the systematic mechanisms of FDY003 activity against BC based on a network pharmacology analysis. FDY003 elicited anticancer effects on human BC cells. Eighteen chemical compounds in FDY003 were identified as potentially bioactive compounds that could target 140 BC-associated genes/proteins and exhibit therapeutic effects. The FDY003 targets were enriched in GO terms associated with the modulation of cellular processes, involving cell proliferation, cell cycle progression, and cell apoptosis. Pathway enrichment analysis of the targets further demonstrated that diverse pathways crucial for the BC pathology were significantly enriched with the FDY003 targets, involving the PI3K-Akt, MAPK, focal adhesion, FoxO, TNF, and estrogen signaling pathways. Based on a network perspective, our findings offer in-depth insights into the therapeutic properties of herbal medicines in BC treatment. Future studies should explore the potential efficacy of the herbal formula in other cancer types as well as its potential efficacy and safety profiles in combination with other therapies.

## Data Availability

The data used to support the findings of this study are included within the article.

## Abbreviations

AcT: *Artemisia capillaris* Thunberg
ADME: Absorption, distribution, metabolism, and excretion
Bax: Bcl-2-associated X protein
C-T: Compound-target
CI: Contribution index
Cm: *Cordyceps militaris*
CTD: The Comparative Toxicogenomics Database
CYP: Cytochrome P450
DL: Drug-likeness
EGFR: Epidermal growth factor receptor
ErbB: Erythroblastic leukemia viral oncogene homolog
FoxO: Forkhead box protein O
GO: Gene ontology
H-C: Herb-compound
H-C-T: Herb-target-pathway
HIF-1: Hypoxia-inducible factor 1
HuGE Navigator: Human Genome Epidemiology Navigator
KEGG: Kyoto Encyclopedia of Genes and Genomes
LjT: *Lonicera japonica* Thunberg
MAPK: Mitogen-activated protein kinase
MTT: 3-(4,5-Dimethylthiazol-2-yl)-2,5-diphenyltetrazolium bromide
NE: Network-based efficacy
OB: Oral bioavailability
OMIM: Online Mendelian Inheritance in Man
PD-1: Programmed cell death protein 1
PD-L1: Programmed death-ligand 1
PharmGKB: Pharmacogenomics Knowledgebase
PI3K: Phosphoinositide 3-kinase
PPI: Protein-protein interaction
QOL: Quality of life
SEA: Similarity ensemble approach
STITCH: Search Tool for Interactions of Chemicals
SysDT: Systematic drug targeting tool
TCMSP: Traditional Chinese medicine systems pharmacology
TTD: Therapeutic Target Database
TNF: Tumor necrosis factor
T-P: Target-pathway
VEGF: Vascular endothelial growth factor
WES algorithm: Weighted ensemble similarity algorithm.

## Supplementary Materials

Supplementary Materials Supplementary Figure S1. Effects of FDY003 on the viability of breast cancer cells. Supplementary Figure S2. Contribution index of active compounds in FDY003. Supplementary Figure S3. Functional enrichment analysis for the breast cancer-associated targets of FDY003. Supplementary Figure S4. Functional interaction analysis for the breast cancer-associated targets of FDY003. Supplementary Table S1. List of the chemical compounds in FDY003. Supplementary Table S2. List of the active chemical compounds in FDY003. Supplementary Table S3. List of the targets of active chemical compounds in FDY003.
