# Supplementary material for: A Network Pharmacology Study on the Molecular Mechanisms of FDY003 for Breast Cancer Treatment
Source: Evid Based Complement Alternat Med. 2021 Feb 6;2021:3919143. doi: 10.1155/2021/3919143 (PMC7881938; doi:10.1155/2021/3919143)
Supplement: Supplementary Materials — Supplementary Figure S1. Effects of FDY003 on the viability of breast cancer cells. Supplementary Figure S2. Contribution index of active compounds in FDY003. Supplementary Figure S3. Functional enrichment analysis for the breast cancer-associated targets of FDY003. Supplementary Figure S4. Functional interaction analysis for the breast cancer-associated targets of FDY003. Supplementary Table S1. List of the chemical compounds in FDY003. Supplementary Table S2. List of the active chemical compounds in FDY003. Supplementary Table S3. List of the targets of active chemical compounds in FDY003. [file 3919143.f1.pdf]

Supplementary Materials

A network pharmacology study on the molecular mechanisms of FDY003 for breast cancer

Ho-Sung Lee[1,2], In-Hee Lee[1], Kyungrae Kang[2], Sang-In Park[3], Seung-Joon Moon[2], Chol Hee Lee[2], and Dae-Yeon Lee[1,2,*]

[1]The Fore, 87 Ogeum-ro, Songpa-gu, Seoul 05542, Republic of Korea.

[2]Forest Hospital, 129 Ogeum-ro, Songpa-gu, Seoul 05549, Republic of Korea.

[3]Forestheal Hospital, 173 Ogeum-ro, Songpa-gu, Seoul 05641, Republic of Korea.

[*]Correspondence should be addressed to Dae-Yeon Lee; foresthrnd@gmail.com

Supplementary Figures

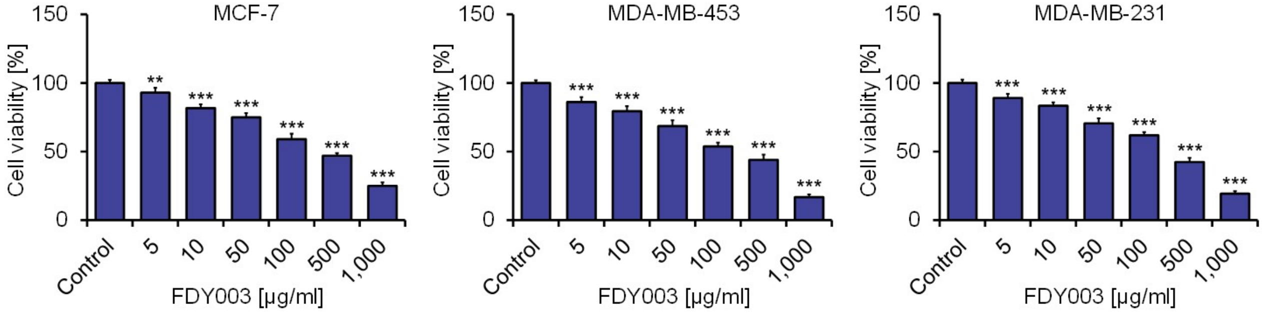

Supplementary Figure S1. Effects of FDY003 on the viability of breast cancer cells. MCF-7, MDA-MB-453, and MDA-MB-231 human breast cancer cells were treated with indicated doses of FDY003 for 72 hours and their viability was measured. Data represent the mean ± S.D. of five replicates. p-values were calculated by comparison with untreated control cells. **, $p < 0.01$; ***, $p < 0.001$; two-tailed Student's t-test.

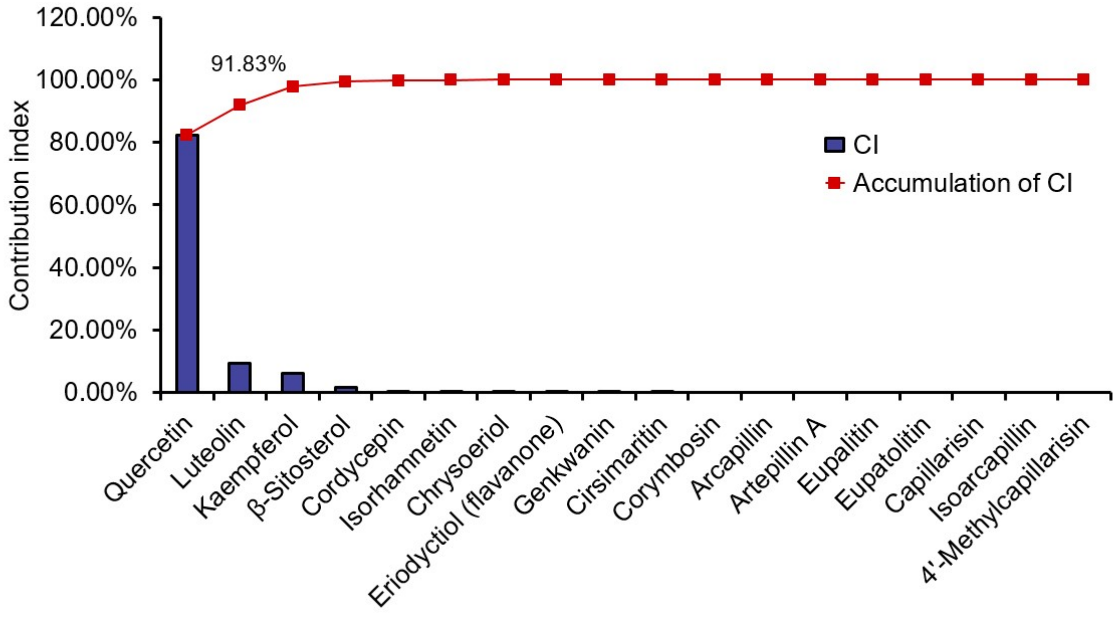

Supplementary Figure S2. Contribution index analysis of active compounds of FDY003. A graph depicting the analysis result of contribution index (CI) of active chemical compounds in FDY003. The sum of CIs for the top two compounds, quercetin and luteolin, was found to exceed 85%.

	Category	Term	Count	Percent [%]	p-value
Gene ontology	Biological process	Cellular aromatic compound metabolic process	82	1.38	7.89E-07
		Cellular hormone metabolic process	16	12.31	8.26E-12
		Cellular response to chemical stimulus	95	2.78	1.42E-33
		Cellular response to chemical stress	28	7.82	4.45E-17
		Cellular response to drug	22	4.91	1.01E-08
		Cellular response to growth factor stimulus	22	3.08	6.48E-05
		Cellular response to hormone stimulus	31	4.28	9.71E-12
		Cellular response to stimulus	115	1.48	2.16E-18
		Cellular response to stress	53	2.52	1.85E-12
		Cellular response to toxic substance	15	5.93	2.42E-06
		Cellular response to vascular endothelial growth factor stimulus	7	10.00	2.33E-03
		Drug metabolic process	29	4.83	3.78E-12
		Estrogen metabolic process	11	34.38	9.27E-13
		Hormone metabolic process	18	7.38	1.20E-09
		Immune response	41	1.79	3.04E-04
		Positive regulation of blood vessel endothelial cell migration	7	8.86	5.31E-03
		Regulation of apoptotic process	53	3.30	1.18E-17
		Regulation of apoptotic signaling pathway	19	4.59	1.02E-06
		Regulation of cell cycle	27	2.13	2.71E-03
		Regulation of cell death	56	3.19	3.03E-18
		Regulation of cell migration	28	2.86	3.18E-06
		Regulation of cell motility	30	2.88	6.45E-07
		Regulation of cell population proliferation	53	3.03	5.72E-16
		Regulation of cellular metabolic process	92	1.44	4.53E-10
		Regulation of cellular response to stress	28	3.66	1.08E-08
		Regulation of defense response	21	2.60	2.18E-03
		Regulation of DNA-binding transcription factor activity	16	3.64	6.41E-04
		Regulation of growth	22	3.17	3.71E-05
		Regulation of hormone levels	27	4.98	1.95E-11
		Regulation of immune system process	40	2.37	1.39E-07
		Regulation of kinase activity	36	3.95	4.77E-13
		Regulation of metabolic process	96	1.37	1.16E-09
		Regulation of mitotic cell cycle phase transition	14	3.13	2.09E-02
		Regulation of programmed cell death	54	3.31	4.01E-18
		Regulation of protein kinase activity	30	3.74	9.70E-10
		Regulation of response to stimulus	80	1.81	2.54E-13
		Regulation of response to stress	45	2.86	6.71E-12
		Regulation of signal transduction	61	1.88	3.40E-09
		Regulation of signaling	72	1.96	5.44E-13
		Regulation of stress-activated protein kinase signaling cascade	10	4.15	3.78E-02
		Regulation of vascular endothelial growth factor production	5	15.63	8.92E-03
		Response to chemical	106	2.16	6.20E-30
		Response to drug	49	4.48	1.17E-21
		Response to estradiol	11	7.80	2.58E-05
		Response to growth factor	22	2.96	1.27E-04
		Response to hormone	40	3.91	1.00E-14
		Response to radiation	21	4.70	7.37E-08
		Response to toxic substance	28	5.09	3.35E-12
		Signal transduction in response to DNA damage	8	5.97	1.97E-02

	Category	Term	Count	Percent [%]	p-value
Gene ontology	Cellular components	Cell	24	2.15	7.02E-04
		Chromosome	28	1.60	2.25E-02
		Cyclin-dependent protein kinase holoenzyme complex	6	14.29	1.93E-04
		Cytoplasm	113	0.97	2.77E-04
		Cytosol	66	1.28	1.26E-04
		Endomembrane system	63	1.39	1.35E-05
		Endoplasmic reticulum	33	1.82	2.71E-04
		Extracellular space	48	1.35	2.90E-03
		Membrane	97	1.00	4.60E-03
		Mitochondrion	26	1.59	4.70E-02
		Nuclear chromosome	24	1.86	8.19E-03
		Organelle	125	0.91	4.13E-04
		Plasma membrane	63	1.12	2.72E-02
		Protein kinase complex	8	7.84	2.81E-04
		Vesicle	51	1.30	4.02E-03
	Molecular function	Aromatase activity	7	25.93	7.64E-07
		Catalytic activity	109	1.86	9.43E-26
		Drug binding	52	2.95	1.36E-15
		Enzyme binding	53	2.36	8.40E-12
		Enzyme regulator activity	21	2.01	3.72E-02
		Estrogen 16-alpha-hydroxylase activity	5	62.50	9.77E-07
		Estrogen 2-hydroxylase activity	3	60.00	3.10E-03
		Kinase activity	37	4.49	7.76E-16
		Ligand-activated transcription factor activity	6	12.77	1.10E-03
		Nuclear receptor activity	6	12.77	1.10E-03
		Protein kinase activity	36	5.87	3.72E-19
		Signaling receptor binding	32	1.95	4.92E-04
		Steroid hormone receptor activity	5	8.93	4.95E-02
		Steroid hydroxylase activity	10	26.32	1.49E-10
		Transcription factor binding	21	3.03	5.60E-05
Pathway	KEGG	Apoptosis	11	8.09	9.38E-04
		Breast cancer	11	7.48	1.96E-03
		Cell cycle	10	8.06	2.45E-03
		Cellular senescence	13	8.23	1.18E-04
		EGFR tyrosine kinase inhibitor resistance	12	15.19	3.23E-07
		Endocrine resistance	17	17.71	1.06E-11
		ErbB signaling pathway	10	11.90	7.17E-05
		Estrogen signaling pathway	12	8.76	1.59E-04
		Focal adhesion	14	7.07	2.80E-04
		FoxO signaling pathway	13	10.16	1.02E-05
		HIF-1 signaling pathway	11	10.09	1.07E-04
		MAPK signaling pathway	15	5.08	6.38E-03
		p53 signaling pathway	9	12.50	1.71E-04
		Pathways in cancer	36	6.81	3.21E-12
		PD-L1 expression and PD-1 checkpoint pathway in cancer	11	12.36	1.34E-05
		PI3K-Akt signaling pathway	24	6.82	1.18E-07
		Platinum drug resistance	7	9.72	1.26E-02
		Ras signaling pathway	12	5.19	2.84E-02
		Steroid hormone biosynthesis	12	20.00	1.14E-08
		TNF signaling pathway	13	11.61	2.03E-06
		VEGF signaling pathway	9	15.25	3.01E-05
		Wnt signaling pathway	10	6.25	2.10E-02

Supplementary Figure S3. Functional enrichment analysis for the breast cancer-associated targets of FDY003. Tables showing the result of gene ontology and pathway enrichment analysis for the breast cancer-related targets of FDY003.

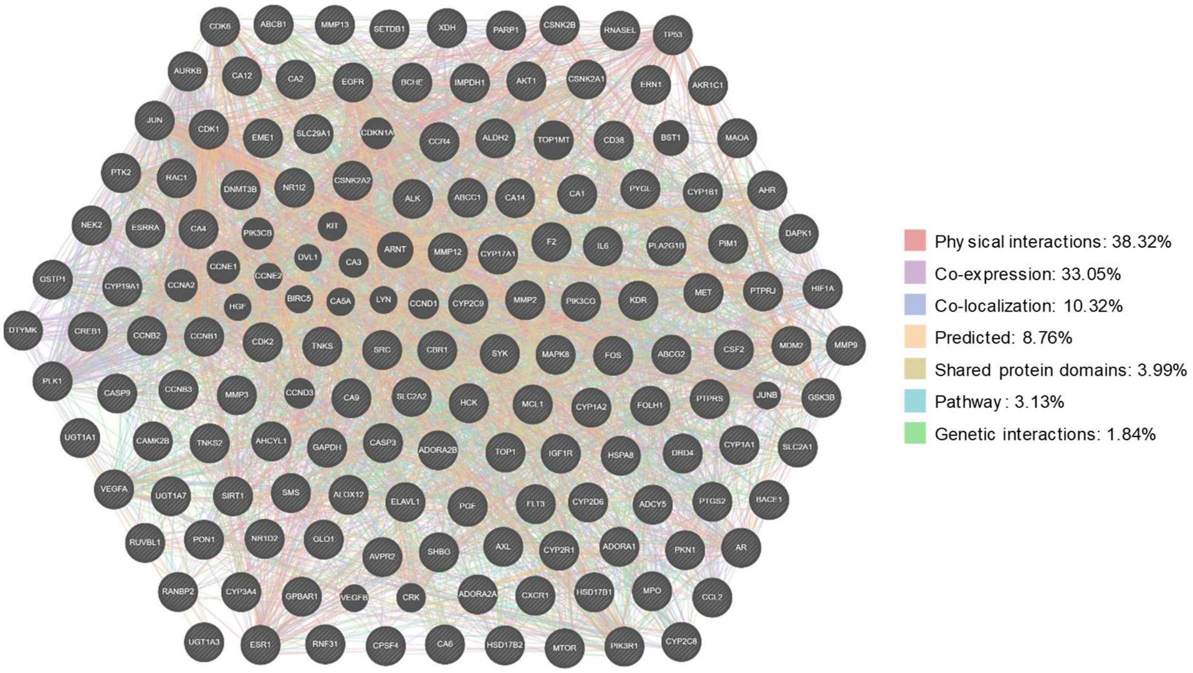

Supplementary Figure S4. Functional interaction analysis for the breast cancer-associated targets of FDY003. Black nodes, breast cancer-related targets; colored edges, distinct mechanisms underlying functional interactions between the targets.

Supplementary Tables

Supplementary Table S1. List of the chemical compounds in FDY003.

Herbal medicines	Compound	OB	Caco-2	DL	Hdon	Hacc	MW	AlogP	RBN
LjT	(-)-(3R,8S,9R,9aS,10aS)-9-ethenyl-8-(beta-D-glucopyranosyloxy)-2,3,9,9a,10,10a-hexahydro-5-oxo-5H,8H-pyrano[4,3-d]oxazolo[3,2-a]pyridine-3-carboxylic acid	3.22	-1.72	0.80	5	12	443.45	-2.71	5
LjT	(-)-(3R,8S,9R,9aS,10aS)-9-ethenyl-8-(beta-D-glucopyranosyloxy)-2,3,9,9a,10,10a-hexahydro-5-oxo-5H,8H-pyrano[4,3-d]oxazolo[3,2-a]pyridine-3-carboxylic acid_qt	87.47	-0.55	0.23	2	7	281.29	-0.96	2
LjT	(-)-Caryophyllene oxide	32.67	1.58	0.13	0	1	220.39	3.52	0
LjT	()-Menthol	59.33	1.27	0.03	1	1	156.30	2.78	1
LjT	(-)-α-Pinene	46.25	1.85	0.05	0	0	136.26	2.87	0
LjT	()-α-Terpineol	46.30	1.28	0.03	1	1	154.28	2.42	1
LjT	(+)-Ledol	16.96	1.43	0.12	1	1	222.41	3.20	0
LjT	(1alpha,3R,4alpha,5R)-3,4,5-Tris[[(2E)-3-(3,4-dihydroxyphenyl)-1-oxo-2-propen-1-yl]oxy]-1-hydroxycyclohexanecarboxylic acid	3.01	-1.46	0.45	7	15	677.63	2.86	13
LjT	(1R,2R,4R)-Dihydrocarveol	51.17	1.32	0.03	1	1	154.28	2.58	1

LjT	(1R,4S,4aR)-1-isopropyl-4-methyl-7-methylene-2,3,4,4a,5,6-hexahydro-1H-naphthalene	19.50	1.84	0.08	0	0	204.39	4.80	1
LjT	(1S,2S)-2-isopropenyl-4-isopropylidene-1-methyl-1-vinylcyclohexane	34.47	1.87	0.06	0	0	204.39	4.93	2
LjT	(1S,4E,8E,10R)-4,8,11,11-tetramethylbicyclo[8.1.0]undeca-4,8-diene	21.69	1.86	0.08	0	0	204.39	4.70	0
LjT	(2S)-2-methylbutan-1-ol	81.23	1.06	0.00	1	1	88.17	1.29	2
LjT	(3R,4aS,5R,6R)-6-hydroxy-3-methoxy-5-vinyl-4,4a,5,6-tetrahydro-3H-pyrano[5,4-c]pyran-1-one	60.89	0.09	0.10	1	5	226.25	-0.04	2
LjT	(5Z,9Z)-6,10,14-trimethylpentadeca-5,9,13-trien-2-one	37.84	1.58	0.10	0	1	262.48	5.45	9
LjT	(E,E,E)-3,7-11,16-tetramethyl hexadeca-2,6,10,14-tetraen-1-ol	42.48	1.42	0.14	1	1	290.54	6.60	11
LjT	(E,Z)-farnesol	36.73	1.32	0.06	1	1	222.41	4.76	7
LjT	(S)-phenethyl 2-bromopropanoate	9.10	1.27	0.05	0	2	257.14	3.19	5
LjT	(Z,E)-farnesol	41.14	1.34	0.06	1	1	222.41	4.76	6
LjT	[(1S)-endo]-(-)-Borneol	83.54	1.22	0.05	1	1	154.28	1.98	0
LjT	1,6-Dicyclohexylhexane	15.40	1.81	0.11	0	0	250.52	7.43	7

LjT	1H,3H-Pyrano(3,4-c)pyran-1-one, 5-ethenyl-6-(beta-D-glucopyranosyloxy)-4,4a,5,6-tetrahydro-, (4aS-(4aalpha,5beta,6alpha))-	4.96	-1.08	0.38	4	9	358.38	-1.59	4
LjT	2-(2,4-dimethoxyphenyl)-3-hydroxy-7-methoxy-chromone	12.94	0.72	0.33	1	6	328.34	2.52	4
LjT	2-(3,4-dimethoxyphenyl)-5-hydroxy-7-methoxy-chromone	29.24	0.90	0.34	1	6	328.34	2.82	3
LjT	2,3-DIMETHYLPYRAZINE	30.82	1.07	0.02	0	2	108.16	0.09	0
LjT	2-[(1R,3S,4S)-3-isopropenyl-4-methyl-4-vinylcyclohexyl]propan-2-ol	19.03	1.37	0.07	1	1	222.41	3.70	3
LjT	2-[(2S,5R)-5-ethenyl-5-methyloxolan-2-yl]propan-2-ol	68.08	0.99	0.04	1	2	170.28	1.43	2
LjT	2-bromododecane	17.60	1.82	0.03	0	0	249.27	5.89	9
LjT	2H-Pyran-5-carboxylic acid, 4-(2,2-dimethoxyethyl)-3-ethenyl-2-(beta-D-glucopyranosyloxy)-3,4-dihydro-, methyl ester, (2S,3R,4S)-	22.59	-1.25	0.45	4	11	434.49	-1.73	10
LjT	2-isopropenyl-5-methylhex-4-enal	27.69	1.33	0.02	0	1	152.26	2.83	4
LjT	2-METHYLPENTADECANE	4.35	1.82	0.06	0	0	226.50	7.47	12
LjT	3,4-Dicaffeoylquinic acid	1.71	-1.40	0.69	6	12	515.48	0.88	9

LjT	3,4-Dimethyl-2-hexanone	35.01	1.38	0.01	0	1	128.24	2.05	3
LjT	3,4-di-O-caffeoylquinic acid methyl ester	1.71	-1.02	0.69	6	12	530.52	1.81	10
LjT	3,5-di-O-caffeoylquinic acid methyl ester	1.73	-0.96	0.68	6	12	530.52	1.81	10
LjT	3-Hexenol	62.74	1.05	0.01	1	1	100.18	1.44	3
LjT	3-Methyl-2-pent-2-enyl-cyclopent-2-enone	25.28	1.52	0.03	0	1	164.27	2.97	3
LjT	3-O-Methylquercetin	10.10	0.20	0.30	4	7	316.28	1.57	2
LjT	4,5-Dicaffeoylquinic acid	1.73	-1.37	0.69	6	12	515.48	0.88	9
LjT	4,5-di-O-caffeoylquinic acid methyl ester	1.73	-0.99	0.69	6	12	530.52	1.81	10
LjT	4,5'-Retro-.beta.,.beta.-Carotene-3,3'-dione, 4',5'-didehydro-	31.22	1.17	0.55	0	2	562.90	9.27	9
LjT	4-caffeoylquinic acid	10.48	-1.52	0.33	5	9	353.33	-1.09	5
LjT	4-stearylmorpholine	14.80	1.48	0.27	0	2	339.68	7.72	17
LjT	5-O-Caffeoyl quinic acid butyl ester	8.77	-0.65	0.41	5	9	410.46	1.16	9
LjT	7-epi-Loganin	4.78	-1.65	0.44	5	10	390.43	-2.08	5
LjT	7-epi-Loganin_qt	85.12	-0.28	0.10	2	5	228.27	-0.33	2

LjT	7-epi-Vogeloside	46.13	-1.30	0.58	4	11	432.47	-0.19	6
LjT	7-epi-Vogeloside_qt	33.26	0.04	0.16	1	6	270.31	0.99	3
LjT	8-epiloganin	11.68	-1.74	0.44	5	10	390.43	-2.08	5
LjT	8-epiloganin_qt	26.42	-0.64	0.10	2	5	228.27	-0.33	2
LjT	9-epi-(E)-caryophyllene	30.28	1.83	0.09	0	0	204.39	4.75	0
LjT	Akebiasaponin D	1.67	-3.02	0.07	11	18	929.23	0.44	10
LjT	Akebiasaponin D_qt	16.44	0.19	0.74	3	4	472.78	5.33	2
LjT	Alloaromadedrene	53.46	1.83	0.10	0	0	204.39	4.22	0
LjT	Amylol	76.16	1.02	0.00	1	1	88.17	1.43	3
LjT	Apigenin	23.06	0.43	0.21	3	5	270.25	2.33	1
LjT	Astragalin	14.03	-1.34	0.74	7	11	448.41	-0.32	4
LjT	Atractylodin	44.49	2.00	0.05	0	1	182.23	3.83	1
LjT	BNL	41.30	1.79	0.04	0	0	166.34	4.69	1
LjT	BZM	18.64	1.36	0.09	0	2	212.26	3.27	4
LjT	C09704	29.56	1.37	0.06	1	1	222.41	4.56	7

LjT	Caeruloside C	55.64	-2.91	0.73	7	15	550.57	-3.70	11
LjT	Caeruloside C_qt	5.40	-1.64	0.37	4	10	388.41	-1.95	8
LjT	Caffeate	54.97	0.27	0.05	3	4	180.17	1.37	2
LjT	CAM	67.17	1.29	0.05	0	1	152.26	1.94	0
LjT	Caprylic acid	16.40	0.90	0.02	1	2	144.24	2.72	6
LjT	Cedrol	16.23	1.35	0.12	1	1	222.41	3.16	0
LjT	Centauroside	4.37	-2.95	0.43	8	19	758.80	-3.11	16
LjT	Centauroside_qt	55.79	-0.84	0.50	2	9	434.48	0.38	10
LjT	Chlorogenic acid	11.93	-1.03	0.33	6	9	354.34	-0.42	5
LjT	Chrysoeriol	35.85	0.39	0.27	3	6	300.28	2.32	2
LjT	CIS-2-PENTENOL	66.40	1.02	0.00	1	1	86.15	1.12	2
LjT	Copaene	29.47	1.81	0.12	0	0	204.39	4.17	1
LjT	Corymbosin	51.96	0.88	0.41	1	7	358.37	2.80	5
LjT	Cosmetin	9.68	-1.08	0.74	6	10	432.41	0.43	4
LjT	D-Camphene	34.98	1.81	0.04	0	0	136.26	2.93	0

LjT	Dehydroxymorroniside	20.69	-1.25	0.46	4	10	388.41	-2.25	5
LjT	Dehydroxymorroniside_qt	29.40	0.12	0.10	1	5	226.25	-0.51	2
LjT	Desaspidinol-A	30.51	0.64	0.05	2	4	182.19	1.02	2
LjT	Dinethylsecologanoside	48.46	-1.37	0.48	4	12	434.44	-0.79	10
LjT	Dinethylsecologanoside_qt	4.50	-0.21	0.12	1	7	272.28	0.39	7
LjT	Disacoside B	1.99	-3.95	0.03	13	22	1075.39	-0.41	12
LjT	Disacoside B_qt	15.83	0.17	0.74	3	4	472.78	5.33	2
LjT	Dodekan	17.74	1.79	0.02	0	0	170.38	5.85	9
LjT	Eriodictyol-7-o-glucoside	17.57	-1.34	0.78	7	11	450.43	0.13	4
LjT	Eriodyctiol (flavanone)	41.35	0.05	0.24	4	6	288.27	2.03	1
LjT	ETHYL FURAN	55.07	1.68	0.01	0	1	96.14	1.75	1
LjT	Ethyl linolenate	46.10	1.54	0.20	0	2	306.54	6.55	15
LjT	Ethylpalmitate	18.99	1.41	0.14	0	2	284.54	6.97	16
LjT	Eudesmol	35.38	1.28	0.09	1	1	224.43	4.10	1
LjT	Eugenol	56.24	1.35	0.04	1	2	164.22	2.55	3

LjT	Farnesene	17.42	1.95	0.05	0	0	204.39	5.52	7
LjT	Farnesol	28.44	1.32	0.06	1	1	222.41	4.76	7
LjT	Farnesol acetate	21.97	1.44	0.11	0	2	264.45	5.14	9
LjT	Flavone der.	27.12	0.83	0.27	1	5	298.31	2.84	3
LjT	Fulvotomentoside A	3.30	-4.60	0.02	15	26	1207.52	-1.65	14
LjT	Geraniol	23.93	1.19	0.02	1	1	154.28	2.93	4
LjT	Germacrene D	19.22	1.83	0.06	0	0	204.39	5.14	1
LjT	Ginnol	11.33	1.46	0.43	1	1	424.89	12.37	26
LjT	GLO	24.44	-1.93	0.03	5	6	180.18	-2.68	5
LjT	Guaiol	38.77	1.36	0.09	1	1	222.41	3.91	1
LjT	Hederagenol	22.42	0.10	0.74	3	4	472.78	5.33	2
LjT	Helixin	9.63	-1.81	0.14	7	12	751.07	3.24	6
LjT	HEPTACOSANE	8.18	1.88	0.36	0	0	380.83	12.69	24
LjT	Heptadecyloxirane	12.44	1.67	0.15	0	1	282.57	7.61	16
LjT	Heptenal	37.16	1.30	0.01	0	1	112.19	2.29	4

LjT	Hexadienal	38.55	1.25	0.01	0	1	96.14	1.39	2
LjT	Hexanal	55.71	1.25	0.01	0	1	100.18	1.85	4
LjT	Hexenal	46.01	1.29	0.01	0	1	98.16	1.83	3
LjT	Hexene	60.01	1.78	0.00	0	0	84.18	2.72	3
LjT	Hyacinthin	38.65	1.31	0.02	0	1	120.16	1.52	2
LjT	Hydnocarpin	2.06	-0.13	0.94	4	9	464.45	3.21	4
LjT	Hydroquinone	29.26	0.89	0.02	2	2	110.12	1.30	0
LjT	HYKOP	32.79	0.25	0.05	3	4	182.19	1.39	3
LjT	Hyperin	6.94	-1.42	0.77	8	12	464.41	-0.59	4
LjT	Indole	34.38	1.81	0.03	1	0	117.16	2.12	0
LjT	Inositol	18.86	-1.52	0.05	6	6	180.18	-3.06	0
LjT	Loniceracetalides B	5.48	-1.44	0.63	4	12	476.53	-0.17	9
LjT	Loniceracetalides B_qt	61.19	-0.09	0.19	1	7	314.37	1.00	6
LjT	Isobutyl tiglate	24.51	1.30	0.02	0	2	156.25	2.76	4
LjT	Isochlorogenic acid C	1.78	-1.32	0.69	7	12	516.49	1.56	9

LjT	Isochlorogenic,acid	1.79	-1.10	0.69	7	12	516.49	1.56	9
LjT	Isothiazole, trimethyl-	67.44	1.42	0.02	0	1	127.23	1.76	0
LjT	Junipene	44.07	1.82	0.11	0	0	204.39	4.18	0
LjT	Kaempferol	41.88	0.26	0.24	4	6	286.25	1.77	1
LjT	Kryptoxanthin	47.25	1.69	0.57	1	1	552.96	10.76	10
LjT	Lauric acid	23.59	1.02	0.04	1	2	200.36	4.54	10
LjT	L-Bornyl acetate	65.52	1.29	0.08	0	2	196.32	2.35	2
LjT	Leukol	35.25	1.59	0.03	0	1	129.17	2.02	0
LjT	Lignoceric acid	14.90	1.24	0.33	1	2	368.72	10.02	22
LjT	Linalool	49.37	0.86	0.04	1	2	170.28	1.43	4
LjT	LINALOOL (D)	38.29	1.29	0.02	1	1	154.28	2.74	4
LjT	Loganic	34.96	-0.58	0.05	4	5	176.19	-0.59	1
LjT	Loganic acid	4.92	-1.81	0.40	6	10	376.40	-2.33	4
LjT	Loganic acid_qt	114.65	-0.59	0.09	3	5	214.24	-0.58	1
LjT	Loganin	5.90	-1.48	0.44	5	10	390.43	-2.08	5

LjT	Loniceracetalide A	28.29	-1.31	0.58	4	11	460.53	-1.16	8
LjT	Loniceracetalide A_qt	89.38	-0.30	0.17	1	6	298.37	0.59	5
LjT	Loniceracetalide B	10.77	-1.37	0.58	4	11	460.53	-1.16	8
LjT	Loniceracetalide B_qt	89.28	0.16	0.17	1	6	298.37	0.59	5
LjT	Luteolin	36.16	0.19	0.25	4	6	286.25	2.07	1
LjT	Luteolin-7-o-glucoside	7.29	-1.23	0.78	7	11	448.41	0.16	4
LjT	Macranthoidin A	4.15	-4.97	0.01	16	27	1237.55	-2.16	15
LjT	Macranthoidin B	6.69	-5.80	0.01	19	32	1399.71	-3.91	18
LjT	Madreselvin A	6.03	-2.50	0.59	10	17	640.60	-2.08	8
LjT	Madreselvin B	3.01	-2.42	0.26	12	20	788.72	-0.36	11
LjT	Mandenol	42.00	1.46	0.19	0	2	308.56	6.99	16
LjT	Methional	53.62	1.04	0.00	0	1	104.19	0.55	3
LjT	methyl (1R,4aS,6S,7R,7aS)-1,6-dihydroxy-7-methyl-1,4a,5,6,7,7a-hexahydrocyclopenta[d]pyran-4-carboxylate	29.99	-0.36	0.10	2	5	228.27	-0.33	2
LjT	Methyl caffeate	30.68	0.54	0.06	2	4	194.20	1.62	3

LjT	Methyl chlorogenate	9.97	-0.74	0.36	5	9	368.37	-0.16	6
LjT	Methyl isomyristate	20.26	1.38	0.08	0	2	242.45	5.50	12
LjT	METHYL LINOLEATE	41.93	1.44	0.17	0	2	294.53	6.64	15
LjT	Methyl myristate	19.68	1.36	0.08	0	2	242.45	5.71	13
LjT	Methyl octadeca-8,11-dienoate	41.93	1.46	0.17	0	2	294.53	6.64	15
LjT	Methyl palmitate	18.09	1.37	0.12	0	2	270.51	6.62	15
LjT	Methyl-9-methyl tetradecanoate	13.70	1.35	0.09	0	2	256.48	5.96	13
LjT	Methyllinolenate	46.15	1.48	0.17	0	2	292.51	6.20	14
LjT	Methyl-p-coumarate	20.14	0.83	0.05	1	3	178.20	1.89	3
LjT	Muurolene	19.50	1.84	0.08	0	0	204.39	4.75	1
LjT	Myristic acid	21.18	1.07	0.07	1	2	228.42	5.46	12
LjT	Neochlorogenic acid	10.65	-1.43	0.33	5	9	353.33	-1.09	5
LjT	Nerol	35.66	1.15	0.02	1	1	154.28	2.93	4
LjT	Neryl acetate	57.47	1.25	0.04	0	2	196.32	3.31	6
LjT	NON	26.74	0.96	0.03	1	2	172.30	3.63	8

LjT	Nonacosanol	10.57	1.48	0.43	1	1	424.89	12.38	27
LjT	Nonanal	40.28	1.31	0.02	0	1	142.27	3.22	7
LjT	Nonanoic acid	40.51	0.92	0.02	1	2	158.27	3.17	7
LjT	Ochnaflavone	2.54	-0.11	0.57	5	10	538.48	4.67	4
LjT	Octadecylglycol	16.18	1.07	0.19	1	2	314.62	7.23	19
LjT	Octanol	21.06	1.16	0.01	1	1	130.26	2.80	6
LjT	Olean-12-en-28-oic acid, 23-hydroxy-3-((O-beta-D-xylopyranosyl-(1-3)-O-6-deoxy-alpha-L-mannopyranosyl-(1-2)-alpha-L-arabinopyranosyl)oxy)-, (3beta,4alpha)-	3.74	-2.34	0.06	9	16	883.20	2.00	8
LjT	Oleanolic acid	29.02	0.59	0.76	2	3	456.78	6.42	1
LjT	o-Thymol	43.28	1.58	0.03	1	1	150.24	3.24	1
LjT	p-Coumaric acid	43.29	0.46	0.04	2	3	164.17	1.64	2
LjT	PEL	44.03	1.11	0.02	1	1	122.18	1.55	2
LjT	Pent-3-en-2-one	50.20	1.25	0.00	0	1	84.13	0.86	1
LjT	Pentadecene	17.72	1.84	0.05	0	0	210.45	6.82	12

LjT	PHB	30.15	0.39	0.03	2	3	138.13	1.17	1
LjT	PHYTANTRIOL	23.20	0.45	0.18	3	3	330.62	5.47	14
LjT	Phytofluene	43.18	2.29	0.50	0	0	543.02	14.10	19
LjT	Prenal	48.87	1.23	0.00	0	1	84.13	1.36	1
LjT	Propyl vinyl ketone	72.27	1.35	0.01	0	1	98.16	1.58	3
LjT	Protocatechuic acid	25.37	0.10	0.04	3	4	154.13	0.90	1
LjT	PTL	59.53	1.21	0.00	0	1	86.15	1.40	3
LjT	Quercetin-3-o-β-D-glu	1.81	-1.40	0.79	8	13	478.39	-0.38	4
LjT	Quinic acid	55.92	-1.79	0.06	4	6	191.18	-3.07	1
LjT	Rhoifolin	6.68	-1.87	0.77	8	14	578.57	-0.43	6
LjT	SCG	23.59	-1.58	0.36	4	10	388.41	-1.98	8
LjT	Scolymoside	3.84	-2.16	0.73	9	15	594.57	-0.70	6
LjT	Secologanate	17.56	-1.90	0.33	5	10	374.38	-2.23	7
LjT	Secologanic acid_qt	73.14	-0.51	0.07	2	5	212.22	-0.49	4
LjT	Secologanic dibutylacetal	20.05	-0.97	0.67	4	11	546.73	1.84	18

LjT	Secologanic dibutylacetal_qt	53.65	0.34	0.29	1	6	384.57	3.58	15
LjT	Secologanin dimethylacetal_qt	2.71	-0.07	0.11	1	6	272.33	0.01	7
LjT	Secologanin_qt	33.33	-0.10	0.08	1	5	226.25	-0.23	5
LjT	Secologanoside	26.92	-2.31	0.37	4	11	388.36	-3.64	7
LjT	Secologanoside 7-methylester	3.88	-1.66	0.45	5	12	420.41	-1.04	9
LjT	Secologanoside 7-methylester_qt	38.01	-0.37	0.11	2	7	258.25	0.13	6
LjT	Secologanoside_qt	79.21	-1.53	0.09	1	6	226.20	-1.89	4
LjT	Secoxyloganin	3.79	-1.63	0.39	5	11	404.41	-2.03	5
LjT	Secoxyloganin_qt	34.80	-0.32	0.09	2	6	242.25	-0.28	1
LjT	Sitogluside	20.63	-0.14	0.62	4	6	576.95	6.34	9
LjT	Stigmasterol	43.83	1.44	0.76	1	1	412.77	7.64	5
LjT	Stigmasterol-β-glucoside	2.40	-0.23	0.63	4	6	574.93	5.89	8
LjT	Succinic acid	29.62	-0.44	0.01	2	4	118.10	-0.41	3
LjT	Sulcatone	26.36	1.35	0.01	0	1	126.22	1.79	3
LjT	Sweroside aglycone	68.68	0.15	0.08	1	4	196.22	0.15	1

LjT	Tetradecane	15.94	1.79	0.04	0	0	198.44	6.76	11
LjT	Thymol	41.47	1.60	0.03	1	1	150.24	3.24	1
LjT	Tricin	27.86	0.51	0.34	3	7	330.31	2.30	3
LjT	Ursolic acid	16.77	0.67	0.75	2	3	456.78	6.47	1
LjT	Vogeloside	5.62	-1.17	0.46	4	10	388.41	-1.78	5
LjT	WLN: NCR B1	55.48	1.61	0.02	0	1	117.16	2.19	0
LjT	WLN: QR BQ DQ	22.93	0.59	0.02	3	3	126.12	1.03	0
LjT	WLN: VHR	32.63	1.32	0.01	0	1	106.13	1.59	1
LjT	XYLOSTOSIDINE	43.17	-1.07	0.64	4	9	415.51	-1.66	4
LjT	XYLOSTOSIDINE_qt	4.01	0.34	0.15	1	4	253.35	0.09	1
LjT	Zeaxanthin	21.17	1.22	0.54	2	2	568.96	9.53	10
LjT	ZINC03978781	43.83	1.32	0.76	1	1	412.77	7.64	5
LjT	Zingiberene	18.80	1.91	0.06	0	0	204.39	5.08	4
LjT	α cadinene	18.73	1.85	0.08	0	0	204.39	4.75	1

LjT	α-Cubebene	16.73	1.83	0.11	0	0	204.39	4.17	1
LjT	β-Carotene	37.18	2.25	0.58	0	0	536.96	12.00	1
LjT	β-Citronellol	38.89	1.20	0.02	1	1	156.30	3.05	5
LjT	β-Cubebene	32.81	1.83	0.11	0	0	204.39	4.22	1
LjT	β-Rhodinol	38.05	1.19	0.02	1	1	156.30	3.05	5
LjT	γ-Muurolene	21.53	1.84	0.08	0	0	204.39	4.80	1
LjT	δ-Amorphene	17.95	1.85	0.08	0	0	204.39	4.94	1
LjT/AcT	Furol	34.35	1.08	0.01	0	2	96.09	0.99	1
LjT/AcT	Heriguard	11.93	-1.03	0.33	6	9	354.34	-0.42	5
LjT/AcT	Quercetin	46.43	0.05	0.28	5	7	302.25	1.50	1
LjT/AcT	Rutin	3.20	-1.93	0.68	10	16	610.57	-1.45	6
LjT/AcT	β-caryophyllene	29.70	1.83	0.09	0	0	204.39	4.75	0
LjT/AcT	β-Elemene	25.63	1.84	0.06	0	0	204.39	4.79	3

LjT/AcT/Cm	β-Sitosterol	36.91	1.32	0.75	1	1	414.79	8.08	6
LjT/Cm	Palmitic acid	19.30	1.09	0.10	1	2	256.48	6.37	14
AcT	(-)-nopinene	44.84	1.80	0.05	0	0	136.26	2.93	0
AcT	(2R,3R)-3,5-dihydroxy-2-(4-hydroxyphenyl)-7-methoxychroman-4-one	24.84	0.14	0.26	3	6	302.30	2.00	2
AcT	(E)-3-[4-hydroxy-3-[(E)-4-hydroxy-3-methyl-but-2-enyl]-5-(3-methylbut-2-enyl)phenyl]acrylic acid	23.76	0.41	0.19	3	4	316.43	4.26	7
AcT	(E)-3-[4-hydroxy-3-[(Z)-4-hydroxy-3-methyl-but-2-enyl]-5-(3-methylbut-2-enyl)phenyl]acrylic acid	12.86	0.35	0.19	3	4	316.43	4.26	7
AcT	(L)-alpha-Terpineol	48.80	1.39	0.03	1	1	154.28	2.42	1
AcT	2-NONANONE	8.51	1.34	0.02	0	1	142.27	2.70	6
AcT	4'-Methylcapillarisin	72.18	0.57	0.35	2	7	330.31	3.06	6
AcT	5-Hydroxyferulate	59.99	0.24	0.07	3	5	210.20	1.35	3
AcT	7-Methylcapillarisin	5.08	0.51	0.34	2	7	330.31	3.06	4
AcT	Arcapillin	48.96	0.60	0.41	3	8	360.34	2.29	4
AcT	Artepillin A	68.32	0.45	0.24	2	4	316.43	3.82	6

AcT	Artepillin C	38.39	0.83	0.17	2	3	300.43	5.35	6
AcT	Ayapanin	41.55	0.97	0.06	0	3	176.18	1.88	1
AcT	Azelex	16.90	-0.04	0.04	2	4	188.25	1.87	8
AcT	Butal	68.66	1.18	0.00	0	1	72.12	0.94	2
AcT	Capillanol	62.02	1.29	0.04	1	1	174.26	2.97	2
AcT	Capillarin	87.01	1.36	0.08	0	2	198.23	3.00	1
AcT	Capillarisin	57.56	0.49	0.31	3	7	316.28	2.81	3
AcT	Capillarol	37.41	0.54	0.12	1	4	260.31	3.17	5
AcT	Capillene	47.19	2.11	0.03	0	0	154.22	3.95	1
AcT	Capillin	52.44	1.50	0.04	0	1	168.20	3.38	1
AcT	Car-3-ene	45.15	1.85	0.04	0	0	136.26	2.87	0
AcT	Cirsilineol	4.81	0.77	0.37	2	7	344.34	2.55	4
AcT	Cirsimaritin	30.35	0.72	0.30	2	6	314.31	2.57	3
AcT	Demethoxycapillarisin	52.33	0.31	0.25	3	6	286.25	2.83	2
AcT	D-limonene	38.80	1.82	0.02	0	0	136.26	3.50	1

AcT	Eugenol	56.24	1.35	0.04	1	2	164.22	2.55	3
AcT	Eupalitin	46.11	0.62	0.33	3	7	330.31	2.01	3
AcT	Eupatolitin	42.55	0.16	0.37	4	8	346.31	1.74	3
AcT	Genkwanin	37.13	0.63	0.24	2	5	284.28	2.59	2
AcT	Hirsutrin	1.86	-1.66	0.77	8	12	464.41	-0.59	4
AcT	Isoarcapillin	57.40	0.40	0.41	3	8	360.34	2.29	4
AcT	Isorhamnetin	49.60	0.31	0.31	4	7	316.28	1.76	2
AcT	Isorhamnetin-3-mono-β-D-glucoside	4.11	-1.35	0.80	7	12	478.44	-0.34	5
AcT	Isorhamnetin-3-O-glucoside	1.17	-1.24	0.80	7	12	478.44	-0.34	5
AcT	Isoscopoletin	23.46	0.71	0.08	1	4	192.18	1.62	1
AcT	Methyleugenol	73.36	1.47	0.04	0	2	178.25	2.81	4
AcT	Neocapillene	24.45	2.09	0.03	0	0	154.22	3.95	0
AcT	Norcapillene	38.06	2.03	0.03	0	0	140.19	4.00	1
AcT	OXL	29.68	-0.64	0.01	2	4	90.04	-0.48	1
AcT	Penta-1,3-diynylbenzene	15.35	2.07	0.03	0	0	140.19	3.50	0

AcT	Piceol	36.80	0.87	0.03	1	2	136.16	1.30	1
AcT	Rhamnocitrin	12.90	0.48	0.27	3	6	300.28	2.02	2
AcT	Salicylic acid	32.13	0.63	0.03	2	3	138.13	1.17	1
AcT	Scoparone	74.75	0.85	0.09	0	4	206.21	1.87	2
AcT	Scopoletin	27.32	0.73	0.08	1	4	192.18	1.62	1
AcT	Scopoletol	27.77	0.71	0.08	1	4	192.18	1.62	1
AcT	Vanillin	52.00	0.68	0.03	1	3	152.16	1.31	2
AcT	α-Humulene	39.81	1.87	0.06	0	0	204.39	5.04	0
Cm	(2R,3R,4S)-2-(6-aminopurin-9-yl)-4-(hydroxymethyl)oxolan-3-ol	38.44	-0.90	0.16	4	7	251.28	-1.48	2
Cm	(2R,3S,5S)-5-(6-aminopurin-9-yl)-2-(hydroxymethyl)oxolan-3-ol	30.13	-1.10	0.15	4	7	251.28	-1.25	2
Cm	20-Hexadecanoylingenol	28.20	0.30	0.68	3	6	586.94	7.38	17
Cm	Adenine	62.81	-0.30	0.03	3	4	135.15	-0.58	0
Cm	Arachidonic acid	45.57	1.20	0.20	1	2	304.52	6.41	14
Cm	Caffeine	89.46	0.58	0.08	0	5	194.22	-0.10	0

Cm	Cerevisterol	39.52	0.35	0.77	3	3	432.76	5.26	4
Cm	Cholesteryl palmitate	31.05	1.45	0.45	0	2	625.19	14.35	21
Cm	Cinnamaldehyde	31.99	1.35	0.02	0	1	132.17	1.95	2
Cm	CLR	37.87	1.43	0.68	1	1	386.73	7.38	5
Cm	Cordycedipeptide A	63.12	-0.34	-	4	6	227.30	-0.65	4
Cm	Cordycepin	36.83	-1.13	-	4	7	251.28	-1.45	2
Cm	Cordylagenin	17.36	0.34	0.78	2	4	432.71	3.66	0
Cm	D-Mannoheptulose	29.74	-2.02	0.05	6	7	210.21	-3.20	6
Cm	EIC	41.90	1.16	0.14	1	2	280.50	6.39	14
Cm	Ergosterol	14.29	1.47	0.72	1	1	396.72	6.93	4
Cm	Galactomannan	10.92	-3.92	0.70	11	16	504.50	-6.01	7
Cm	GLB	47.71	-1.89	0.04	5	6	180.18	-2.51	1
Cm	GUP	43.04	-1.82	0.04	5	6	180.18	-2.51	1
Cm	Isoergotamine	8.10	0.32	0.21	3	9	581.73	3.12	4
Cm	LFA	8.46	1.83	0.13	0	0	282.62	9.50	17

Cm	Linoleic	41.90	1.23	0.14	1	2	280.50	6.39	14
Cm	Linoleyl acetate	42.10	1.36	0.20	0	2	308.56	6.85	16
Cm	MTL	17.73	-1.58	0.03	6	6	182.20	-2.94	5
Cm	NCA	71.13	0.44	0.02	2	3	122.14	-0.32	1
Cm	Nicotinic acid	47.65	0.34	0.02	1	3	123.12	0.28	1
Cm	Oleic acid	33.13	1.14	0.14	1	2	282.52	6.84	15
Cm	Peroxyergosterol	44.39	0.86	0.82	1	3	428.72	6.73	4
Cm	Stearic acid	17.83	1.15	0.14	1	2	284.54	7.28	16
Cm	Styrone	38.35	1.14	0.02	1	1	134.19	1.69	2
Cm	TGL	15.13	0.54	0.13	0	6	891.67	22.26	56
Cm	Thiamine	19.87	-0.32	0.11	3	4	265.40	-0.05	4
Cm	TRE	2.32	-3.08	0.24	8	11	342.34	-4.26	4
Cm	Uracil	42.53	0.05	0.02	2	4	112.10	-1.01	0
Cm	Uralene	11.70	0.63	0.49	4	7	384.41	3.43	4
Cm	Uridine	10.49	-1.14	0.11	4	8	244.23	-2.45	2

Cm	Vitamin C	13.34	-0.86	0.04	4	6	176.14	-1.76	2
Cm	Vitamin G	6.79	-1.22	0.50	5	10	376.41	0.23	5

LjT, *Lonicera japonica* Thunberg; AcT, *Artemisia capillaris* Thunberg; Cm, *Cordyceps militaris*; MW, molecular weight; OB, oral bioavailability; Caco-2, Caco-2 cell permeability; DL, drug-likeness score; Hdon, number of hydrogen bond donors; Hacc, number of hydrogen bond acceptors; MW, molecular weight; AlogP, octanol-water partition coefficient log P; RBN, number of rotatable bonds.

Supplementary Table S2. List of the active chemical compounds in FDY003.

Herbal medicines	Compound	OB	Caco-2	DL	Hdon	Hacc	MW	AlogP	RBN
LjT	Chrysoeriol	35.85	0.39	0.27	3	6	300.28	2.32	2
LjT	Corymbosin	51.96	0.88	0.41	1	7	358.37	2.80	5
LjT	Eriodyctiol (flavanone)	41.35	0.05	0.24	4	6	288.27	2.03	1
LjT	Loniceracetalides B_qt	61.19	-0.09	0.19	1	7	314.37	1.00	6
LjT	Kaempferol	41.88	0.26	0.24	4	6	286.25	1.77	1
LjT	Luteolin	36.16	0.19	0.25	4	6	286.25	2.07	1
LjT/AcT	Quercetin	46.43	0.05	0.28	5	7	302.25	1.50	1
LjT/AcT/Cm	β-Sitosterol	36.91	1.32	0.75	1	1	414.79	8.08	6
AcT	4'-Methylcapillarisin	72.18	0.57	0.35	2	7	330.31	3.06	6
AcT	Arcapillin	48.96	0.60	0.41	3	8	360.34	2.29	4
AcT	Artepillin A	68.32	0.45	0.24	2	4	316.43	3.82	6
AcT	Capillarisin	57.56	0.49	0.31	3	7	316.28	2.81	3
AcT	Cirsimaritin	30.35	0.72	0.30	2	6	314.31	2.57	3

AcT	Demethoxycapillarisin	52.33	0.31	0.25	3	6	286.25	2.83	2
AcT	Eupalitin	46.11	0.62	0.33	3	7	330.31	2.01	3
AcT	Eupatolitin	42.55	0.16	0.37	4	8	346.31	1.74	3
AcT	Genkwanin	37.13	0.63	0.24	2	5	284.28	2.59	2
AcT	Isoarcapillin	57.40	0.40	0.41	3	8	360.34	2.29	4
AcT	Isorhamnetin	49.60	0.31	0.31	4	7	316.28	1.76	2
Cm	Cordycepin	36.83	-1.13	-	4	7	251.28	-1.45	2

LjT, *Lonicera japonica* Thunberg; AcT, *Artemisia capillaris* Thunberg; Cm, *Cordyceps militaris*; MW, molecular weight; OB, oral bioavailability; Caco-2, Caco-2 cell permeability; DL, drug-likeness score; Hdon, number of hydrogen bond donors; Hacc, number of hydrogen bond acceptors; MW, molecular weight; AlogP, octanol-water partition coefficient log P; RBN, number of rotatable bonds.

Supplementary Table S3. List of the targets of active chemical compounds in FDY003.

Herbal medicines	Compound	Targets
LjT	Chrysoeriol	ABCC1*, CREB1*, CYP1B1*, PTPRS*, XDH*
LjT	Corymbosin	ABCG2*, CREB1*, CYP1B1*, PTPRS*
LjT	Eriodyctiol (flavanone)	ABCC1*, CA4*, CA7, CA12*, CBR1, CYP1A2*, CYP1B1*, CYP3A4*, CYP19A1*, HSD17B1*, PGF*, SHBG*, TAS2R31*
LjT	Kaempferol	ABCB1*, ABCC1*, ABCG2*, AHR*, AKR1B1, ALOX5, AR*, BCHE*, CA2*, CA7, CA12*, CDK1*, CISD1, CTDSP1, CYP1A2*, CYP1B1*, CYP2D6*, DAPK1*, EGFR*, ESR1*, ESRRA*, F2*, FLT3*, HCK*, HSD17B1*, HSD17B2*, MPO*, NOX4, NR1I2*, P4HB, PIM1*, PTPRS*, SLC2A1*, TYR, UGT3A1, XDH*
LjT	Luteolin	ABCG2*, ADORA1*, AKR1B1, AKR1B10, ALOX5, ALOX15, APP, ARG1, CA2*, CA4*, CA7, CA12*, CASP3*, CCNB1*, CCNB2*, CCNB3*, CD38*, CDK1*, CDK2*, CDK5R1, CDK6*, CREB1*, CSNK2A2*, CSNK2B*, CYP1A2*, CYP1B1*, FLT3*, FOS*, GLO1*, GPR35, GSK3B*, JUN*, MAOA*, MAPK8*, MMP12*, MMP2*, MMP9*, NOX4, PARP1*, PTPRS*, SYK*, TNKS*, TNKS2*, TOP1*, TTR, XDH*
LjT/AcT	Quercetin	ABCB1*, ABCC1*, ABCG2*, ADORA1*, ADORA2A*, AKR1A1, AKR1B1, AKR1C1*, AKR1C2, AKR1C3, AKR1C4, AKT1*, ALK*, ALOX5, ALOX12*, ALOX15, AR*, ATP5A1, ATP5B, ATP5C1, AURKB*, AVPR2*, AXL*, BACE1*, CA1*, CA2*, CA3, CA4*, CA5A, CA6*, CA7, CA9*, CA12*, CA13, CA14*, CAMK2B*, CASP3*, CASP9*, CCL2*, CCR4*, CDK1*, CSF2*, CSNK2A1*, CXCR1*, CYP1A1*, CYP1A2*, CYP1B1*, CYP2C8*, CYP2C9*, CYP3A4*, CYP19A1*, DAPK1*, DRD4*, EGFR*, ELAVL1*, F2*, FLT3*, GLO1*, GPR35, GSK3B*, GSTP1*, HCK*, HIBCH, HIF1A*, HSD17B2*, IGF1R*, IL6*, JUN*, KDR*, MAOA*, MAPK8*, MCL1*, MET*, MMP2*, MMP3*, MMP9*, MMP13*, MPO*, NEK2*, NEK6, NOX4, NUAK1, P4HB, PIK3CG*, PIK3R1*, PIM1*, PKN1*,

		PLA2G1B*, PLK1*, PON1*, PTGS2*, PTK2*, PTPRS*, PYGL*, SIRT1*, SLC2A2*, SRC*, STK17B, TP53*, UGT1A1*, UGT1A3*, UGT1A7*, UGT3A1, VEGFA*, XDH*
LjT/AcT/Cm	β-Sitosterol	ACBD7, CYP17A1*, DHPS, DRAP1, GPBAR1*, LFT, MDM2*, MEX3D, MTOR*, NPC1L1, NR1D2*, RAC1*, RANBP2*, RUVBL1*, TNNC1, USF1
AcT	4'-Methylcapillarisin	CREB1*
AcT	Arcapillin	CREB1*, CYP1B1*, PTPRS*
AcT	Artepillin A	CPSF4*, CYP2R1*, EME1*, FOLH1*, PTPRJ*, RNF31*
AcT	Capillarisin	CREB1*, CYP1B1*, PTPRS*
AcT	Cirsimaritin	ADORA1*, ADORA2A*, ADORA3, AKR1B1, CREB1*, CYP1B1*, PTPRS*
AcT	Eupalitin	CREB1*, CYP1B1*
AcT	Eupatolitin	CREB1*, CYP1B1*
AcT	Genkwanin	ALDH2*, CREB1*, CYP1B1*, PTPRS*, XDH*
AcT	Isoarcapillin	CREB1*, PTPRS*
AcT	Isorhamnetin	CA2*, CA4*, CA7, CA12*, CREB1*, CYP1A1*, CYP1B1*, ERN1*, P4HB, XDH*
Cm	Cordycepin	ADCY5*, ADK, ADORA1*, ADORA2A*, ADORA2B*, ADORA3, AHCY, AHCYL1*, AHCYL2, AMD1, CASP9*, DCK, DGUOK, DNMT3B*, DOT1L, DTYMK*, GAPDH*, HSPA5, HSPA8*, IMPDH1*, KMT5C, MTAP, P2RX1, P2RY11, PRMT7, QARS1*, RARS, RNASEL*, SETDB1*, SLC29A1*, SMS*, SRM, TARS1*, TK1

LjT, *Lonicera japonica* Thunberg; AcT, *Artemisia capillaris* Thunberg; Cm, *Cordyceps militaris*.

*, Breast cancer-associated targets.
